# Face‐touching behaviour as a possible correlate of mask‐wearing: A video observational study of public place incidents during the COVID‐19 pandemic

**DOI:** 10.1111/tbed.14094

**Published:** 2021-05-18

**Authors:** Lasse S. Liebst, Peter Ejbye‐Ernst, Marijn de Bruin, Josephine Thomas, Marie R. Lindegaard

**Affiliations:** ^1^ Department of Sociology University of Copenhagen Copenhagen Denmark; ^2^ The Netherlands Institute for the Study of Crime and Law Enforcement Amsterdam Netherlands; ^3^ Radboud University Medical Center Radboud Institute of Health Sciences Amsterdam Netherlands; ^4^ Corona Behavioural Unit National Institute for Public Health and the Environment (RIVM) Amsterdam Netherlands; ^5^ Department of Sociology University of Amsterdam Amsterdam Netherlands

**Keywords:** COVID‐19, face‐touching, masks, real‐life behaviour, video observation

## Abstract

Most countries in the world have recommended or mandated face masks in some or all public places during the COVID‐19 pandemic. However, mask use has been thought to increase people's face‐touching frequency and thus risk of self‐inoculation. Across two studies, we video‐observed the face‐touching behaviour of members of the public in Amsterdam and Rotterdam (the Netherlands) during the first wave of the pandemic. Study 1 (*n* = 383) yielded evidence in favour of the absence of an association between mask‐wearing and face‐touching (defined as touches of face or mask), and Study 2 (*n* = 421) replicated this result. Secondary outcome analysis of the two studies—analysed separately and with pooled data sets—evidenced a negative association between mask‐wearing and hand contact with the face and its t‐zone (i.e. eyes, nose and mouth). In sum, the current findings alleviate the concern that mask‐wearing has an adverse face‐touching effect.

## 1 INTRODUCTION

Mask‐wearing in some or all public settings has been widely used as a preventive measure to mitigate the spread of the coronavirus disease‐2019 (COVID‑19). One concern regarding the use of face masks among members of the public is that mask wearers may adjust or otherwise touch their mask, thus creating a potential hand‐to‐face route for the virus to enter the body through the nose, eyes, and mouth (i.e. the t‐zone) (ECDC, 2020; West et al., 2020; WHO, 2020a).

Fortunately, recently available evidence offers some reassurance, with epidemiological research showing a low risk of COVID‐19 surface contamination (Mondelli et al., 2020) and behavioural observational studies reporting either no association (Perez‐Alba et al., 2020; Tao et al., 2020) or a negative association (Chen et al., 2020; Kungurova et al., 2020; Lucas et al., 2020; Shiraly et al., 2020) between mask‐wearing and face‐touching. For example, in the most extensive study published to date, Chen et al. (2020) video observed 7,586 members of the public across East Asia, Western Europe and North America, before and during the coronavirus pandemic. They report an overall negative association—especially with respect to t‐zone touching—although this association was only significant in the East‐Asian contexts. This latter null result may, however, reflect a reliance on small subsamples, with very few mask wearers. Here, we offer an analysis of the association between mask‐wearing and face‐touching in an additional Western European context, applying a sample with a more balanced number of masked and unmasked persons. To this end, we pre‐registered an analysis plan (available at osf.io/bj7tg) and tested the hypothesis in two studies that mask‐wearing is negatively associated with face‐touching.

## 2 STUDY 1

### 2.1 Materials and methods

Data comprised video footage of real‐life public behaviour in Amsterdam, the Netherlands, captured by public security cameras during the COVID‐19 pandemic, between May and the beginning of June 2020 (data and materials are available at osf.io/7ek9d). Data were recorded over 5 days (three Thursdays, one Saturday and one Sunday) between the morning hours and the evening. We obtained data from the Amsterdam police with the permission of the Attorney General at the Ministry of Public Affairs. The raw sample comprised more than 30,000 hr of raw footage recorded across around 50 cameras. From this sample, we selected footage from a single camera (to eliminate potential between‐context heterogeneity), which satisfied the following inclusion criteria: The camera should have a high recording quality and allow for a detailed observation of the pedestrians with only negligible breaks in the coverage. Further, to counter the circumstance that public security cameras are disproportionately found in densely populated settings (Philpot et al., 2019), the camera should capture a street as average as possible (in this case, a pedestrianized street located outside the touristy inner‐city). Finally, we assessed all footage and excluded intervals with technical recording issues.

#### 2.1.1 Coding procedure

Two trained student research assistants coded data in accordance with a behavioural codebook (‘ethogram’) that we developed with inspiration from ethologists studying animal and human behaviour (Eibl‐Eibesfeldt, 1989; Jones et al., 2016). This involved descriptive classifications of recurrent behaviours as observed in the natural, public environment. As part of this procedure, we also adapted and assessed the ecological validity of prior behavioural definitions (e.g. face‐touching) found in the literature (Kwok et al., 2015). To ensure the epidemiological validity of the face‐touching measures, we consulted an infectious disease specialist at The National Institute for Public Health and the Environment (the Netherlands).

The coding began by splitting the eligible footage into 30 min segments and then randomly selecting 51 of these. We planned to sample seven masked and seven unmasked persons for each segment (in practice, we could not always satisfy this criterion because of too few mask wearers per segment). Mask‐wearing included individuals wearing respirators (e.g. N95), surgical masks or fabric masks. We excluded persons wearing face shields, eye protection, improvised masks (e.g. bandana, scarf), and persons wearing masks covering neither the nose nor the mouth. We also excluded persons who put the mask on or took it off, or changed the mask's placement in the face (e.g. from covering both nose and mouth to mouth only). As such, the current data offer insights into the face‐touching rate related to one part of the behavioural sequence involved in face mask use (Von Cranach, 1982)—that is, the ‘subconscious’ face‐touching (e.g. fidgeting, scratching) when mounted rather than ‘deliberate’ mask repositioning (Hall et al., 2007; Perl et al., 2020).^1^

We observed each person for the duration captured on camera walking through the study setting (although for a maximum of two minutes). The average observation time per individual was 25 s (*SD* = 7.42), with a total of 158 person‐minutes of observation. In sum, we sampled 176 persons wearing a face mask and 207 not wearing one, comprising a total sample size of 383 persons. This sample size satisfies an a priori statistical power analysis suggesting that 339 observations would detect a small effect (f^2^ = 0.05), with a power of 90% and a conservative alpha of 0.005 (Benjamin et al., 2018). Note that we coded beyond what the power analysis suggested to have a buffer for incomplete cases (the decision to terminate the sampling procedure was taken before any analyses were conducted). For the inter‐rater reliability test of the codebook, we selected 44 individuals and 25 contexts for independent double coding, with a Krippendorff’s (2004) alpha (α) larger than 0.8 as a benchmark for acceptable agreement (each score is reported below).

#### 2.1.2 Measures

The primary face‐touching outcome was captured as a binary variable distinguishing between whether or not the person touched his or her face or a potential mask at least once with the hand (α = 0.89) (for illustrations of the face‐touching measures see osf.io/7ek9d). This definition aligned with the recommendation that appropriate mask use involves that neither the face nor the front of the mask should be touched (WHO, 2020b). Note that individuals who used hand sanitizer were disqualified from being recorded as a positive touching events.

Further, we included three secondary measures with a more narrow definition of face‐touching, capturing direct hand‐to‐face contacts only (i.e. for these outcomes, touching the mask was defined as a non‐event). These additional measures captured direct hand contact with the face, the mid‐face, or the t‐zone. The ‘face’ was defined as including eyes, nose, mouth, ears, cheeks, chin and forehead (α = 0.87). The ‘mid‐face’ was restricted to the area from the top of the brows to the tip of the chin with the width of the jaw (α = 1.0), and the ‘t‐zone’ included the eyes, nostrils and mouth (α = 0.50). Note that a low incident rate of the t‐zone measure entailed an unreasonably low α score despite a high percentage of between‐coder agreement (98%). Gwent's (2008) AC1 is considered a more robust inter‐rater statistic for such heavily skewed variables (i.e. t‐zone touches were rare), and this test yielded an excellent score of 0.98.

The independent face mask variable captured whether the person wore a face mask covering the nose and mouth, or either the nose or the mouth (α = 1.00). We also included a number of controls: visual assessments of the persons’ age (α = 0.86) and gender (α = 0.95), the number of seconds the person was observed (α = 0.94), and the level of people crowding of the 30 min segment from where the person was sampled (α = 1.00).^2^

#### 2.1.3 Estimation

Data were estimated with linear probability models (using Stata 16’s ‘reg’ module) (Breen et al., 2018), specified with cluster‐robust standard errors to account for the hierarchical data structure (i.e. individuals nested in 30 min segments). Given the insight that the traditional alpha level of 0.05 offers a weak evidential threshold (Colquhoun, 2017), we followed the recommendation to evaluate alpha levels of 0.05 and 0.005 as ‘suggestive’ and ‘significant’, respectively (Benjamin et al., 2018). All reported *p*‐values are two‐tailed. Besides *p*‐values, we report Bayes factors (BFs) approximated from Bayesian information criteria (assuming a unit‐information prior), which allow for quantification of evidence in favour of the absence of an association (Wagenmakers, 2007).

### 2.2 Results

Table 1 presents the summary statistics of the study samples. The primary (face or mask touching) outcome was suggestively less common in Study 1 than in Study 2 (*p* = .006, Fisher's exact test). No between‐study differences were found with respect to the secondary outcomes of direct hand touching of the face (*p* = .067, Fisher's exact test), the mid‐face (*p* = .384, Fisher's exact test) or the t‐zone (*p* = .877, Fisher's exact test). Further, there was no between‐study difference in the gender composition (*p* = .105, Fisher's exact test). Compared with Study 1, Study 2 had a lower age average (*t*(804) = 3.8,*p* < .001), was more crowded (*t*(804) = −5.3,*p* < .001), included a larger proportion of persons in company with some else (*p* < .001, Fisher's exact test) and had a higher average temperature (*t*(804) = −10.6,*p* < .001).

**TABLE 1:** Summary statistics

	Study 1		Study 2		Combined analysis	
	*M*	*SD*	*M*	*SD*	*M*	*SD*
Mask‐wearing	0.46	0.50	0.39	0.49	0.43	0.49
Primary outcome						
face or mask touching	0.13	0.33	0.20	0.40	0.16	0.37
Secondary outcomes						
face‐touching	0.10	0.30	0.14	0.35	0.12	0.33
mid‐face‐touching	0.08	0.27	0.10	0.30	0.09	0.28
t‐zone touching	0.05	0.27	0.5	0.22	0.05	0.23
Controls						
Age	39.09	14.61	35.31	13.75	37.11	14.28
Male gender	0.48	0.50	0.53	0.50	0.51	0.50
People crowding	13.99	7.21	18.22	14.21	16.21	11.62
Observation duration	24.77	7.42	23.31	17.84	24.00	13.91
Additional controls						
Avg. temperature	18.15	4.26	21.49	4.67	19.91	4.78
Together with someone	0.23	0.42	0.59	0.49	0.42	0.49
Mask mandate zone	‐	‐	0.34	0.47	0.18	0.38

**FIGURE 1:**
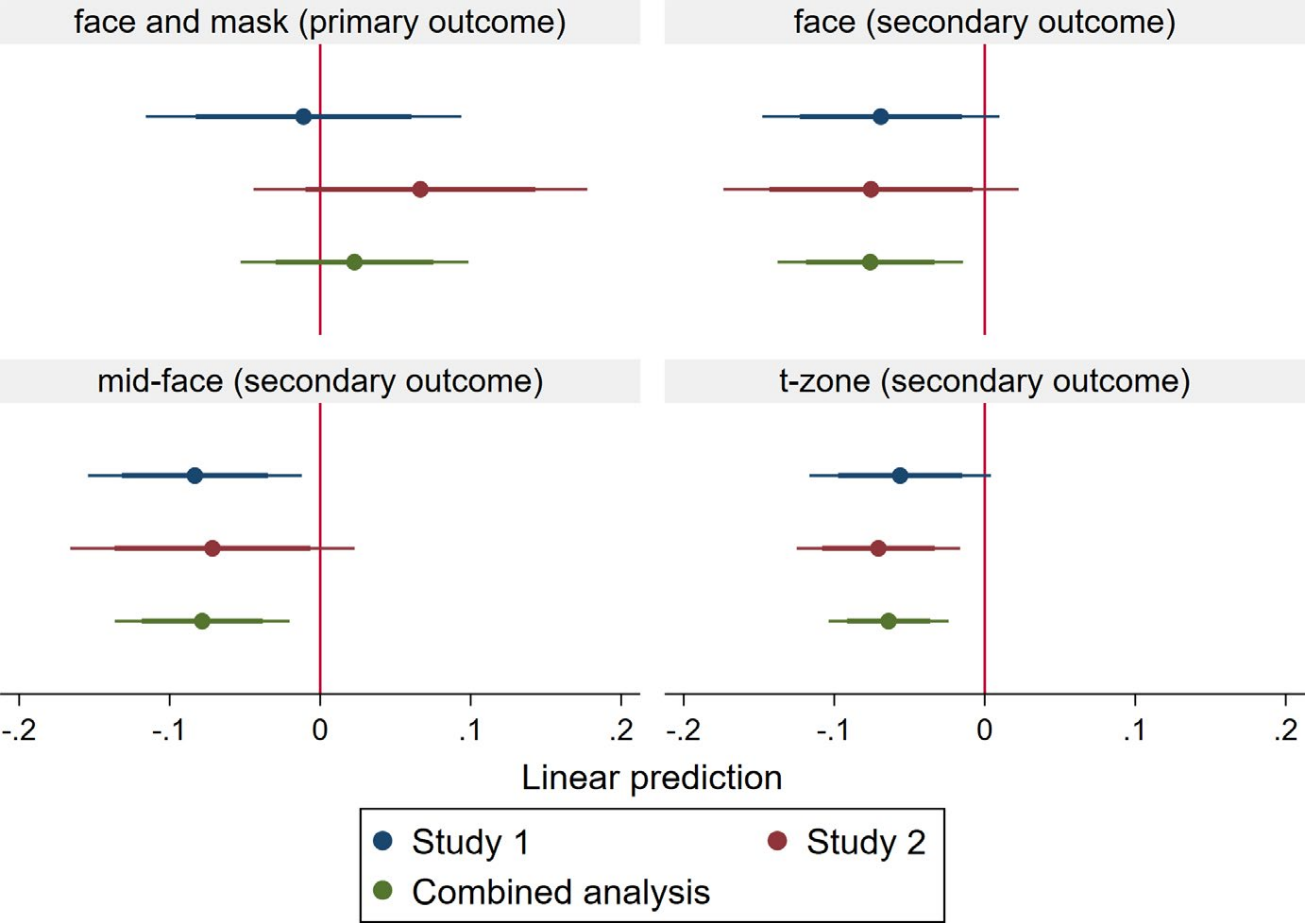
Regression results of the association between mask‐wearing and the primary and secondary face‐touching outcomes in Study 1, Study 2 and Combined analysis. Note Beta estimates and 95% and 99.5% confidence intervals, controlled for age, gender, observation duration and people crowding (for estimated results of the control variables, see the full regression output at osf.io/7ek9d)

Across the average person‐observation time of around 25 s, 12% (21/176) of the masked and 13% (27/207) of the unmasked persons touched their face or mask (primary outcome). As seen in Figure 1, contrary to our hypothesis, a regression analysis of this association found no evidence of a link between mask‐wearing and the primary outcome, B = −0.01, CI 95% [−0.08, 0.06], *p* = .759, BF_01_ = 18.57. The Bayes factor suggested that the *H_0_* was around 19 times more likely than *H*_a_, which may be considered substantial‐to‐strong evidence in favour of the absence of an association (Raftery, 1995).

Next, regarding the secondary outcomes, 6% (11/176) of the masked and 13% (27/207) of the unmasked persons touched their face, 3% (6/176) of the masked and 12% (24/207) of the unmasked persons touched the mid‐face, and 2% (4/176) of the masked and 8% (17/207) of the unmasked persons touched their t‐zone. In line with our hypothesis, these figures indicate that mask‐wearing was linked with a lower frequency of face‐touching, and the regression results confirmed this: Mask‐wearing was negatively associated with direct hand contact with the face (β = −0.07, CI 95% [−0.12, −0.02], *p* = .013, BF_01_ = 1.57), the mid‐face (β = −0.08, CI 95% [−0.13, −0.03], *p* = .001, BF_10_ = 4.89), and the t‐zone (β = −0.06, CI 95% [−0.10, −0.01], *p* = .009, BF_01_ = 1.08). The Bayes factor evidence also evidenced this with respect to mid‐face‐touching, while data could not discriminate *H*_0_ and *H*_1_ with respect to direct face and t‐zone touches. In terms of practical significance, these results indicate that mask‐wearing was linked with around 6–8 percentage points lower probability for direct hand‐to‐face contacts. That is a small effect size—equivalent to a Cohen’s (1988) *d* at around between −0.20 and −0.30—although the effect may cumulate across time (Funder & Ozer, 2019). In sum, these results offer suggestive evidence for a negative association between masks and face‐touching, although the robustness of the evidence hinges on how face‐touching was operationalized.

## 3 STUDY 2

### 3.1 Materials and methods

Study 2 was designed as a replication of Study 1. There are a few noteworthy between‐study differences, however. Specifically, data for Study 2 were collected as part of a larger research project evaluating the implementation of mandatory mask‐wearing zones in Amsterdam and Rotterdam. Across these contexts, we collected footage from six comparable locations (rather than from a single camera, located in Amsterdam, as in Study 1), three of which had an operative mask mandate. The common denominator of the areas was that they were above‐average busy pedestrianized streets—as also reflected in the circumstance that Study 2 was more crowded than Study 1. Across the included locations, data were selected during 13 days (Wednesdays, Saturdays and one Sunday) between late July and the end of August 2020. From a raw sample of around 480 hr of footage, we randomly selected 78 30 min segments, across which we conducted 164 person‐minutes of observation, with an average observation time of 23 s (*SD* = 17.84). Applying the reliability‐tested codebook from Study 1, a team of 12 trained student research assistants coded 423 persons (167 masked and 256 unmasked).

### 3.2 Results

Across the average person‐observation time of 23 s, 23% (38/167) of the masked and 18% (46/254) of the unmasked persons touched their face or mask. As we see in Figure 1, a regression analysis found no association between mask‐wearing and this primary (face or mask touching) outcome (β = 0.07, CI 95% [−0.01, 0.14], *p* = .086, BF_01_ = 4.74), with the related Bayes factor offering substantial evidence in favour of the absence of an association.

Regarding the secondary outcomes, 8% (14/167) of the masked and 18% (46/254) of the unmasked persons touched their face, 5% (8/166) of the masked and 13% (33/255) of the unmasked persons touched the mid‐face and 0.60% (1/166) of the masked and 8% (21/255) of the unmasked persons touched their t‐zone. In line with these descriptive patterns, we found suggestive regression evidence in favour of negative association between mask‐wearing and direct face touches of the face (β = −0.08, CI 95% [−0.14, −0.01], *p* = .029, BF_01_ = 1.67) and mid‐face (β = −0.07, CI 95% [−0.14, −0.01], *p* = .032, BF_01_ = 1.08), although the corresponding Bayes factors suggest that data could not discriminate between *H*_0_ and *H*_1_. With respect to the third secondary outcome—t‐zone touching—we found statistically significant and substantial Bayes factor evidence for a negative association with mask‐wearing, β = −0.07, CI 95% [−0.11, −0.03], *p* < .001, BF_10_ = 7.54. The effect sizes of mask‐wearing on the secondary outcomes are small in magnitude, similar to Study 1. In sum, both the primary and secondary outcome analyses of Study 2 replicate the overall results of Study 1, with data suggesting that mask‐wearing was non‐associated with the primary (face or mask touching) outcome and negatively associated with the secondary (direct hand‐to‐face contact) outcomes.

## 4 COMBINED ANALYSIS

### 4.1 Materials and methods

A prospect of the two current data sets is that they may be pooled into one large and high‐powered dataset (Cooper & Patall, 2009). Such combined (‘mega’) analysis is a more appropriate approach to synthesize results across the studies than to simply ‘tally‐vote’ positive, negative, and null findings (Hedges & Olkin, 1980). Further, given its added statistical power, it is also plausible that such pooled and highly powered analysis allows for a more accurate estimation of the associations reported in Study 1 and 2 (Gelman & Carlin, 2014; Maxwell et al., 2008).^3^

We combined this approach with an explorative assessment of how robust the link between mask‐wearing and face‐touching is across (the ‘multiverse’ of) other plausible data and model specifications (Steegen et al., 2016). In total, we estimated 12,288 unique model specifications (using the ‘mrobust’ module by Young, 2018), including all possible combinations of the following features: First, the Study 1 and Study 2 data sets analysed separately and pooled. Second, the primary and secondary outcomes. Third, the four independent variables, including three additional ones: (a) whether mask‐wearing was mandatory or voluntary in the location; (b) whether the person was alone or together with someone; (c) and the temperature of each 30 min segment.^4^ Fourth, whether the mask covered both nose and mouth, or only one of these areas. Fifth, in‐ and exclusion of persons relocating or putting their mask on/took. Finally, estimation of data with linear and logistic models.

### 4.2 Results

As we see in Figure 1, the pooled analysis offered a less ambiguous and arguably more accurate picture of the patterns found in Study 1 and 2. The association between mask‐wearing and the primary outcome remained non‐significant, again with the Bayes factor offering evidence for a non‐association, β = 0.02, CI 95% [−0.03, 0.08], *p* = .391, BF_01_ = 19.38. Also, we found statistically significant and substantial‐to‐strong Bayes factor evidence for negative associations between mask‐wearing and the secondary outcomes capturing direct touches of the face (β = −0.08, CI 95% [−0.12, −0.03], *p* = .001, BF_10_ = 8.62), the mid‐face (β = −0.08, CI 95% [−0.12, −0.04], *p* < .001, BF_10_ = 65.34), and the t‐zone (β = −0.06, CI 95% [−0.09, −0.04], *p* < .001, BF_10_ = 94.92).

Next, the robustness analysis across the 12,288 specifications added further credence to these findings: In more than nine out of ten models specified with one of the secondary outcomes, the association was negative and below an alpha level of 0.05. More specifically, for models specified with either face, mid‐face, or t‐zone touching as the outcome, we found a negative association in 100%, 98% and 92% of the models, respectively. This contrasts the subset of models specified with the primary (face and mask touching) outcome, in which only 4.7% yielded a suggestive, positive association. Furthermore, when assessed with a conservative 0.005 alpha threshold, 0% of the models specified with the primary outcome remained significant. The models specified with the secondary outcomes were comparatively more (although not uniformly) robust, with 47%, 65% and 63% significantly negative estimates with respect to models specified with the face, mid‐zone and t‐zone outcome, respectively.^5^

## 5 DISCUSSION

The wide use of face masks as a measure against the coronavirus disease‐2019 raises the question of whether mask‐wearing by the general public is linked with adverse behavioural effects (ECDC, 2020; Mantzari et al., 2020), including an increased face‐touching frequency. The current paper showed that face‐touching is a fairly common occurrence and tested the hypothesis that mask‐wearing is linked with less face‐touching. Initially, contrary to this hypothesis, our analysis of the primary face‐touching outcome in Study 1 and 2 both found evidence in favour of a non‐association. However, our secondary outcomes analysis found that the association with mask‐wearing hinges on how face‐touching is operationalized—a common but underappreciated experience in statistical research (Silberzahn et al., 2018; Steegen et al., 2016). Specifically, in line with our hypothesis, Study 1 and 2 and the Combined analysis found relatively robust negative correlations of mask‐wearing with the secondary outcomes measuring direct hand‐to‐face contacts.

Our findings correspond with the prior studies, which either report no association (e.g. Tao et al., 2020) or a negative association (e.g. Lucas et al., 2020), especially with respect to t‐zone touches (Chen et al., 2020). Taken as a whole, current and prior evidence alleviate the concern that mask‐wearing has a positive and adverse face‐touching effect (WHO, 2020a). The absence of such an effect may be ascribed to how face masks serve as a physical barrier for direct hand‐to‐face contact or offer a reminder that face‐touching should be avoided (see Latour, 1999).

One limitation of the current paper is how generalizable our results—based on cross‐sectional data from video‐monitored public spaces in two Dutch cities—are to other countries, settings or pandemic phases. In particular, we were restricted by the localization of the security cameras in outdoor public settings where people are mainly passing through. For example, it is plausible that mask‐wearing in semi‐public and indoor settings with more sociable exchanges—a potential facilitator of self‐touching gestures (Streeck, 2020)—may yield other face‐touching outcomes. Another limitation is that the question at hand concerns a causal process—do face masks impact face‐touching behaviours—while our observational approach merely conveys correlational insights. Due to the lack of experimental control, it may be that unobserved factors, rather than the face mask itself, underpin the negative association with face‐touching (e.g. persons who choose to wear a mask may be more careful not to touch their face).

A further potential limitation relates to our focus on face‐touching occurring during one step of the behavioural sequence involved in mask use, that is, while the mask is in place rather than when it is put on, off, or relocated. Highlighting this point, the few positive associations found in the Combined analysis with respect to the primary outcome were found in models including the (otherwise excluded) persons who relocated or put their mask on/off. However, this link is not surprising nor necessarily problematic, but may simply be seen as an artefact of how the primary outcome defines mask touches (along with face touches) as a positive event—that is, the mask is unavoidably touched when it is replaced. Whether the face is also directly touched as part of such mask replacement is a critical question that should be systematically addressed in future research (in the current data, 2 in 10 persons moving their mask also touched their face directly).

Finally, it should be acknowledged that while naturalistic observation of public human behaviour has high ecological validity, this method is an often‐messy experience involving many ad hoc decisions that may challenge the study's replicability. This is illustrated by how current results hinged on how face‐touching was defined. To counteract this and similar measurement issues, future research should prioritize the development of reproducible standards via open sharing of codebooks, data, and—if it can be done ethically—raw videos (Gilmore & Adolph, 2017).

## CONFLICT OF INTEREST

The authors declare no conflicts of interest.

## ETHICAL APPROVAL

The authors confirm that the ethical policies of the journal, as noted on the journal's author guidelines page, have been adhered to. This work was approved by the Ethics Committee for Legal and Criminological Research at Vrije University. Further, it complies with the American Psychological Association's ethics code (APA, 2010: Section 8.03), stating that analysis of image data recorded in public places does not require informed consent from those recorded.

## Data Availability

The data, scripts, and materials that support the findings of this study are openly available at osf.io/7ek9d.

## ENDNOTES

^1^
Note that this exclusion of (in total four) persons who repositioned or put the mask on/off was not part of the initial codebook of Study 1, and, as such, deviates from our pre‐registration (see osf.io/bj7tg and an exclusion flow chart at osf.io/7ek9d). However, this was done to align Study 1 with Study 2 where this exclusion selection criterion was introduced, because this mask behaviour proved to be disproportionately common in this context. This reflects the area‐based mask mandate operative in part of the Study 2 context, potentially skewing the sample towards this particular mask behaviour. We evaluate the statistical and conceptual implications of this exclusion in the study limitation section and in the Combined analysis (for this assessment, we relied on the four persons originally sampled in Study 1 and additional six persons from Study 2, who happened to be sampled despite their exclusion from sampling procedure). Note that the decision to exclude these persons was done before any analyses were run.

^2^
In Study 1, we counted all pedestrians and bicycles passing through an imaginary ‘gate’ in both directions twice for two minutes per segment, and then calculated the average number of people crossing per minute. To make the coding procedure more time efficient, we only counted the number of crossing persons for one minute per segment in Study 2.

^3^
Specifically, a sensitivity power analysis suggested that the minimal detectable effect of the pooled analysis was f^2^ = 0.02, that is what is often considered the lower threshold of a small‐sized effect (Cohen, 1988). As such, a sample size larger than the pooled data set would only allow for a potential detection of an effect of limited practical significance (Kirk, 1996).

^4^
The temperature measures were constructed using publicly available data from the Royal Netherlands Meteorological Institute (KNMI). The measurement of whether the person was alone or together with someone had an inter‐rater score of 0.95.

^5^
Note that we explored (across 1,024 models) whether the control variables included in Study 1 and 2 and the Combined analysis were associated with the primary outcome and the secondary outcomes. None robust associations were found see (see osf.io/7ek9d), except (and obviously) the observation duration (i.e. the longer a person was observed, the more likely it was to record a face‐touching event).
